# Survival time tool to guide care planning in people with dementia

**DOI:** 10.1212/WNL.0000000000008745

**Published:** 2020-02-04

**Authors:** Miriam L. Haaksma, Maria Eriksdotter, Debora Rizzuto, Jeannie-Marie S. Leoutsakos, Marcel G.M. Olde Rikkert, René J.F. Melis, Sara Garcia-Ptacek

**Affiliations:** From the Department of Geriatric Medicine (M.L.H., M.G.M.O.R., R.J.F.M.), Radboudumc Alzheimer Center, Radboud Institute for Health Sciences, Radboud University Medical Center, Nijmegen, the Netherlands; Aging Research Center (M.L.H., D.R.), Department of Neurobiology, Care Sciences and Society, Karolinska Institutet, Solna; Division of Clinical Geriatrics (M.E., S.G.-P.), Department of Neurobiology, Care Sciences and Society, Karolinska Institutet; Theme Aging (M.E., S.G.-P.), Karolinska University Hospital, Huddinge, Sweden; Department of Psychiatry (J.-M.S.L.), Division of Geriatric Psychiatry and Neuropsychiatry, Johns Hopkins University School of Medicine, Baltimore, MD; Radboud University Medical Center (M.G.M.O.R.), Donders Institute for Brain, Cognition and Behaviour, Department of Geriatric Medicine, Radboudumc Alzheimer Center, Nijmegen, the Netherlands; and Department of Internal Medicine (S.G.-P.), Section for Neurology, Södersjukhuset Stockholm, Sweden.

## Abstract

**Objective:**

To develop survival prediction tables to inform physicians and patients about survival probabilities after the diagnosis of dementia and to determine whether survival after dementia diagnosis can be predicted with good accuracy.

**Methods:**

We conducted a nationwide registry-linkage study including 829 health centers, i.e., all memory clinics and ≈75% of primary care facilities, across Sweden. Data including cognitive function from 50,076 people with incident dementia diagnoses ≥65 years of age and registered with the Swedish Dementia Register in 2007 to 2015 were used, with a maximum follow-up of 9.7 years for survival until 2016. Sociodemographic factors, comorbidity burden, medication use, and dates of death were obtained from nationwide registries. Cox proportional hazards regression models were used to create tables depicting 3-year survival probabilities for different risk factor profiles.

**Results:**

By August 2016, 20,828 (41.6%) patients in our cohort had died. Median survival time from diagnosis of dementia was 5.1 (interquartile range 2.9–8.0) years for women and 4.3 (interquartile range 2.3–7.0) years for men. Predictors of mortality were higher age, male sex, increased comorbidity burden and lower cognitive function at diagnosis, a diagnosis of non-Alzheimer dementia, living alone, and using more medications. The developed prediction tables yielded c indexes of 0.70 (95% confidence interval [CI] 0.69–0.71) to 0.72 (95% CI 0.71–0.73) and showed good calibration.

**Conclusions:**

Three-year survival after dementia diagnosis can be predicted with good accuracy. The survival prediction tables developed in this study may aid clinicians and patients in shared decision-making and advance care planning.

Dementia is a neurodegenerative syndrome accompanied by increased morbidity and mortality.^1–3^ The prevalence of dementia is expected to increase because of longer life expectancies around the globe; the proportion of people dying with or due to dementia also will grow.^4^

*The Lancet* Commission on Dementia Prevention, Intervention and Care has emphasized the importance of discussing the future with patients and their families and considering the needs and wishes of patients toward the end of life.^4^ Clinical guidelines also recommend incorporating information on patients' life expectancy into clinical decisions.^5^ However, clinicians appear to encounter several barriers in this process.^6,7^ One of the barriers for the incorporation of patient's life expectancy in clinical decisions is the uncertainty in predicting the actual survival probabilities. Another barrier is the difficulty of discussing prognosis with the patient.^6^ The uncertainty in predicting survival probabilities is caused partly by the lack of a proper prediction model.

Discussing a patient's prognosis can be facilitated by a clear tool that visualizes patients' prognosis on the basis of their personal and clinical characteristics. Timely communication about patients' survival prognosis may enhance advance care planning and shared decision-making in dementia. In the present study, we focus on patients with a diagnosis of dementia from the Swedish Dementia Registry (Svenska Demensregistret [SveDem]). The aims of this study were to predict their survival using routinely collected patient characteristics and to develop risk tables that can be used to improve the communication of patients' prognosis in clinical practice.

## Methods

### Data sources

This cohort study is based on patients registered with SveDem from May 2007 to December 2015. SveDem (svedem.se) is a national quality registry for monitoring the diagnosis, treatment, and care of people with dementia in Sweden.^8^ In December 2015, SveDem included a total of 58,154 individuals meeting the ICD-10 criteria for dementia.^9^ All patients in our cohort are people with incident dementia diagnoses from either primary care or specialist memory clinics. The Swedish Death Registry was used to obtain dates of death for patients up until August 28, 2016. This registry is maintained by Statistics Sweden and covers 100% of all deaths in Sweden.^10^ The Swedish Prescribed Drug Register was used to obtain information on drug use. All prescribed drugs provided through the pharmacy were recorded in this register. The Swedish National Patient Register was used to obtain medical diagnoses of patients in the cohort. This register contained medical diagnoses from inpatient and specialized outpatient visits in Sweden from 2000 to 2014, classified according to the ICD-10 criteria. Both the National Patient Register and the Prescribed Drug Register are maintained by the National Board of Health and Welfare; together, they cover >99% of all inpatient medical diagnoses and expedited drugs.^11,12^

### Assessment of demographic, medical, and medication data

Demographic data at the moment of diagnosis were obtained from SveDem and included age, sex, living situation (living at home alone, living at home with a coresident, or institutionalized), Mini-Mental State Examination (MMSE) score (range 0–30),^13^ and dementia type. Dementia diagnoses were coded as Alzheimer disease (AD), vascular dementia, mixed dementia (AD and vascular dementia), dementia with Lewy bodies, frontotemporal dementia, Parkinson disease dementia, unspecified dementia, and other dementia types. A detailed description of the diagnostic criteria can be found elsewhere.^8^ The number of expedited drugs during the 3 months before dementia diagnosis was extracted from the Swedish Prescribed Drug Register. The medical diagnoses up until the moment of dementia diagnosis were selected from the Swedish National Patient Register and used to calculate the Charlson Comorbidity Index (CCI) score (range 0–16).^14^

### Study population

We selected people with late-onset dementia, that is, persons who were ≥65 years of age at the time of dementia diagnosis (n = 55,578). Patients who lacked information on explanatory variables (n = 5,487; 9.9%) or follow-up time (n = 15) were excluded, yielding a total of 50,076 patients. A flowchart of the sample selection process is available from Dryad (figure e-1, doi.org/10.5061/dryad.p46tr17). Missing explanatory variables were MMSE score (n = 2,618), living situation (n = 668), or dementia type in the memory clinic setting (n = 2,525). Compared to included patients, patients who were excluded due to missing values scored lower on the MMSE (Δ = −0.93 point, *p* < 0.001) and used more drugs (Δ = 0.38, *p* < 0.001). Although the median survival was somewhat shorter among the excluded patients (median survival time 4.3 [interquartile range (IQR) 2.3–6.9] years compared to 4.8 [IQR 2.6–7.6] years in the included cohort, log-rank *p* < 0.001), they did not differ from the included patients in terms of age, CCI score, and sex.

### Statistical analysis

To predict survival, we used routinely collected characteristics measured at the moment of diagnosis. These included age, sex, dementia type, living situation, MMSE score, CCI score, and number of drugs used in the 3 months before diagnosis. Survival time was measured beginning on the date of diagnosis. The last recorded date of death (August 28, 2016) was used as a censoring date for survivors. Kaplan-Meier estimates were used to assess the median and IQR of survival times in different population strata. Cox proportional hazards models were used to calculate hazard ratios (HRs) with 95% confidence intervals (CIs) for mortality.^15^ The assumption of proportionality was verified by examining log minus log plots and Schoenfeld residuals. The different dementia diagnoses were grouped according to their HR: types that did not differ significantly from each other (i.e., with similar HRs) were combined. Dementia subtype was often unspecified for patients diagnosed in primary care settings; hence, we built separate prediction models for patients from primary care and memory clinics. In the models for patients diagnosed in memory clinics, dementia subtype was included as a predictor of survival, whereas this covariate was excluded in the models for primary care patients. A forward selection procedure was used to select significant predictors of survival (*p* < 0.05). To derive a practical tool from the prediction models, we created risk tables showing the 3-year survival probabilities for several combinations of predictor values (i.e., risk factor profiles). To provide a concise yet discriminatory and accurate tool, we used the order of entering from the forward selection procedure to select the 5 strongest predictors for inclusion in our risk tables.

We assessed the performance of our models by examining the discrimination and calibration.^16^ To assess the discriminative ability of the model, the Harrell c index (i.e., a concordance statistic) was calculated. This index ranges from 0 to 1, with higher values denoting a higher ability of the model to discriminate between patients who survived and those who died. To determine the prognostic accuracy of the model, the agreement between predicted and observed survival curves was visually assessed with calibration plots. To assess the internal validity of our developed models, we calculated shrinkage factors and the optimism in the Harrell c indexes through bootstrap cross-validation (150). To compare included patients with those who were excluded due to missing values, we used 1-sample *t* tests and χ^2^ tests. Statistical analyses were conducted with SAS version 9.4 (SAS Institute Inc, Cary, NC), supplemented by the macros survcstd*,*^17^ calibrationplots, and OptimismShrinkageCox.

### Standard protocol approvals, registrations, and patient consents

Ethics permission for this study was obtained from the regional human ethics committee of Stockholm (2015/743-31/4). Quality registries such as SveDem are considered an important part of the development and improvement of health and social care in Sweden. Each patient has to be informed about the registration and has the right to decline participation. Written consent is not required; however, each patient has the right to obtain a copy of the information that is registered if requested and to withdraw consent.

### Data availability

Requests for access to the SveDem data should be addressed to the registry holder and the steering committee (svedem.se).

## Results

### Sample characteristics

A total of 50,076 patients from 829 Swedish health centers were included in the analysis, with a maximum follow-up time of 9.7 years. Median age at diagnosis was 81.6 (IQR 76.5–86.0) years, and the median time to death was 4.8 (IQR 2.6–7.6) years. The most common dementia type was AD (n = 15,945, 31.8%), followed by mixed dementia (n = 10,226, 20.4%) and vascular dementia (n = 10,040, 20.0%). There were 10,298 patients with an unspecified dementia type (20.6%). The majority of the sample was female (59.4%) and living at home (90.4%). People diagnosed in primary care were older (Δ = 3.0, *p* < 0.001), were more often female (χ^2^ = 111.4, *p* < 0.001), and had a lower MMSE score (Δ = −0.8, *p* < 0.001) compared to people diagnosed in memory clinics. The sample characteristics at the moment of diagnosis are summarized in table 1.

**Table 1:**
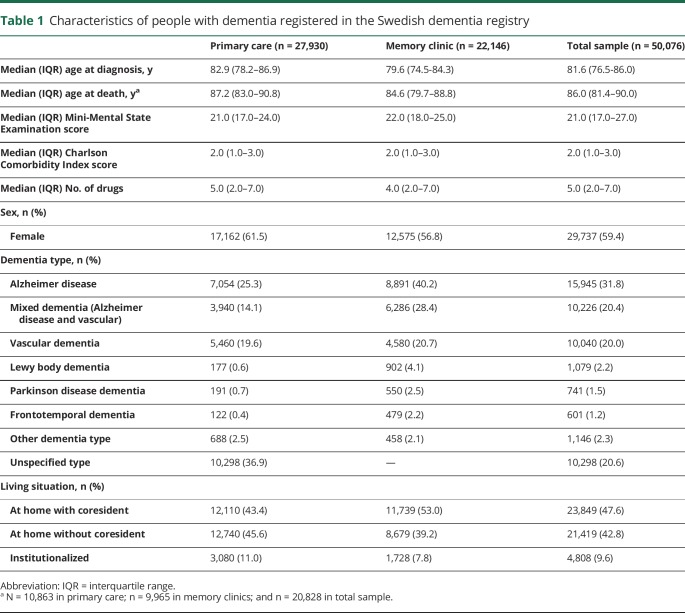
Characteristics of people with dementia registered in the Swedish dementia registry

### Predictors of survival

After 9.7 years of follow-up, 20,828 patients (41.6%) had died (10,863 patients from primary care and 9,965 patients from the memory clinic setting). The estimated median survival time from diagnosis of dementia was 4.8 (IQR 2.6–7.6) years. Survival times stratified by risk factors for mortality are shown in table 2.

**Table 2:**
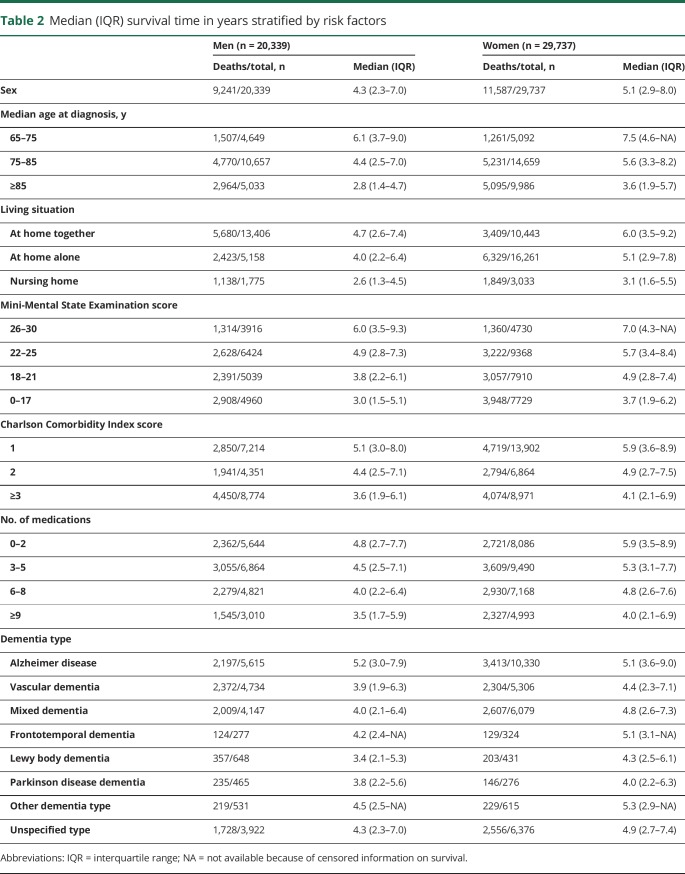
Median (IQR) survival time in years stratified by risk factors

All included covariates depicted in table 2 were significant predictors of survival in univariable analyses and in the multivariable models. Moreover, patients diagnosed at memory clinics generally showed higher mortality risk compared to those diagnosed in primary care (HR 1.15, 95% CI 1.11–1.19) after correction for all included covariates. The results of the multivariable Cox regression models for survival across the entire follow-up period are depicted in table 3. Log minus log plots and Schoenfeld residuals showed no deviation from the proportional hazards assumption.

**Table 3:**
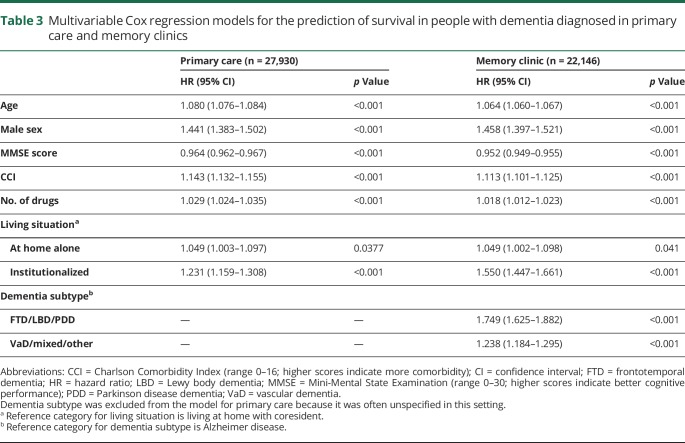
Multivariable Cox regression models for the prediction of survival in people with dementia diagnosed in primary care and memory clinics

**Figure 1:**
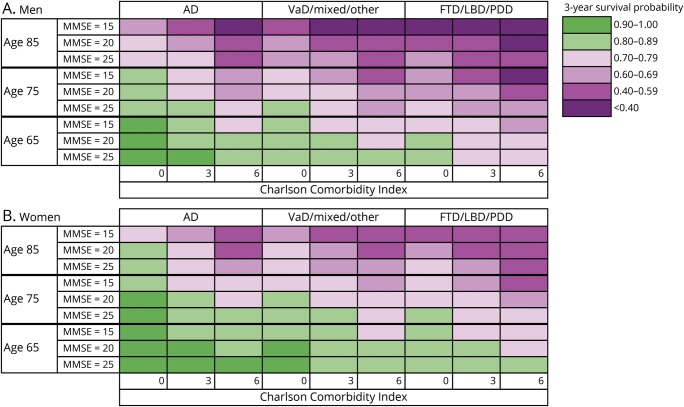
Three-year survival probabilities for (A) men and (B) women with dementia diagnosed in a memory clinic AD = Alzheimer disease; FTD = frontotemporal dementia; LBD = Lewy Body Dementia; MMSE = Mini-Mental State Examination; PDD = Parkinson disease dementia; VaD = vascular dementia.

**Figure 2:**
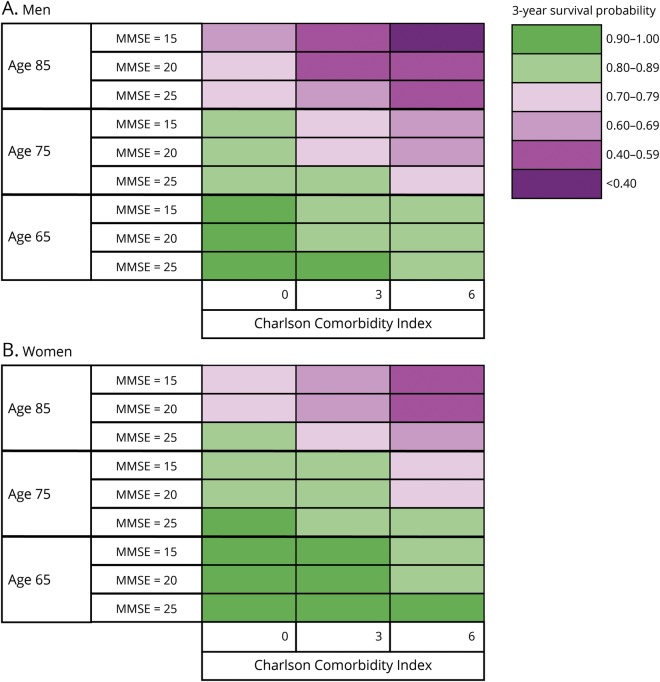
Three-year survival probabilities for (A) men and (B) women with dementia diagnosed in primary care MMSE = Mini-Mental State Examination.

In the model for memory clinic patients, predictors were included in the following order by the forward selection procedure: age, global cognition, comorbidity, dementia subtype, sex, living situation, and number of drugs. The order of inclusion was the same for the model based on primary care patients except that comorbidity was included before global cognition and dementia subtype was not included in this model. There was a significant interaction between living situation and sex in our model for primary care patients. While living alone was a significant risk factor for mortality in men, this was not so for women. Forward selection of predictors showed that age, sex, comorbidity, and global cognition were the strongest predictors of survival in both diagnostic settings, in addition to dementia subtype for patients diagnosed at a memory clinic. To visualize the impact of these predictors on survival, risk tables were created for male and female patients from primary care and memory clinics. These tables show the 3-year survival probabilities for several risk factor profiles (memory clinic, figure 1; primary care, figure 2). For example, a 75-year-old patient with AD from a memory clinic who scores 25 on the MMSE and 3 on the CCI has a 3-year survival probability of 0.85 (95% CI 0.83–0.86). Standard errors of our estimated 3-year survival probabilities were all <0.04. Although significant in the multivariable model, the last 2 predictors included by the forward selection procedure, i.e., living situation and number of drugs, added little discriminatory value to our prediction model. This is shown by table 4, which lists the c indexes of models with increasing numbers of variables. The increase in c index after the last 2 selection steps was very small. To provide a concise yet discriminatory and accurate tool, the variables living situation and number of drugs were not included in the figures. The HRs of our final models are shown in table 5.

**Table 4:**
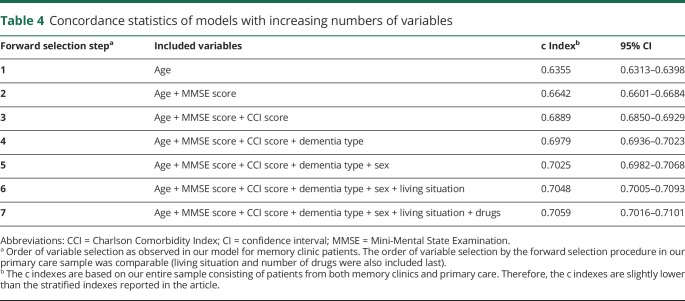
Concordance statistics of models with increasing numbers of variables

**Table 5:**
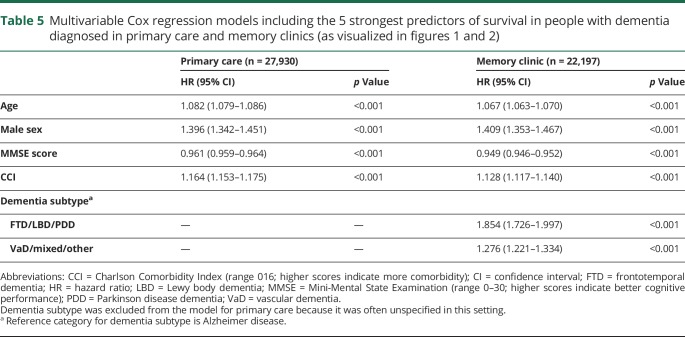
Multivariable Cox regression models including the 5 strongest predictors of survival in people with dementia diagnosed in primary care and memory clinics (as visualized in figures 1 and 2)

### Model performance

To evaluate the ability of our risk tables to discriminate between those who survived the first 3 years after diagnosis and those who did not, we calculated the c index on the basis of the predictors included in figures 1 and 2 (5 predictors for patients from a memory clinic, 4 predictors for patients from primary care). The c index of the model for memory clinic patients was 0.71 (95% CI 0.70–0.72) for men and 0.72 (95% CI 0.71–0.73) for women. The c index of the model for primary care patients was 0.70 (95% CI 0.69–0.71) for men and 0.71 (95% CI 0.70–0.71) for women. This is substantially higher than the c index based on age and sex only, which was 0.65 (95% CI 0.64–0.65). The calibration plots of the models on which our tables were based showed strong agreement between predicted and observed survival curves, indicating good accuracy of our prediction (figures e-2 and e-3 available from Dryad, doi.org/10.5061/dryad.p46tr17). Bootstrapped cross-validation procedures revealed shrinkage factors between 0.994 and 0.996 and optimism in the Harrell c index <0.001 for all of the 4 models on which the risk tables were based.

## Discussion

This study shows that it is possible to provide accurate 3-year survival probabilities for patients with dementia in this nationwide Swedish patient registry on the basis of 5 (4 for primary care setting) characteristics obtained at diagnosis: age, sex, comorbidity status, cognitive performance, and dementia type. Mortality risk increased with increasing age, morbidity, and cognitive impairment. In accordance with the general population, in our study, men had a lower life expectancy than women. Moreover, patients with AD had a higher life expectancy compared to patients with non-AD dementia types. After correction for covariates, patients diagnosed at the memory clinic generally showed higher mortality risk compared to those diagnosed in primary care. The reason probably is that patients with more complex disease patterns (e.g., non-AD dementia with faster disease progression or more severe comorbidity) are more likely to be referred to a memory clinic. For this reason, we choose to stratify our results by diagnosis setting. This stratification reflects actual clinical practice because dementia subtype is often unspecified in patients diagnosed in primary care. The observed mean survival time was 5.1 (standard error 0.02) years in our cohort with a mean age of 81.1 years at diagnosis of dementia. In comparison, the average 80-year-old person in Sweden has a life expectancy of 9 years.^18^ This average is based on the general Swedish population, which includes a significant proportion of persons with dementia, so it should be noted that average survival for persons who do not develop dementia is expected to be even longer. These results are very similar to previously reported numbers from a UK population study^19^ and fit with our current knowledge of the detrimental effect of dementia on life expectancy.

SveDem is the world's largest cohort of people with dementia, including all memory clinics and ≈75% of primary care facilities across Sweden. Therefore, this is one of the largest studies to date examining survival in dementia. Strengths compared to the previous SveDem study on survival^20^ are the inclusion of patients diagnosed in primary care, comprehensive information on comorbidity burden, and more exact information on the number of medications. Moreover, by converting HRs into risk tables with 3-year survival probabilities, we have created one of the first tools to estimate survival of people with incident dementia diagnoses and to inform decision-making in clinical practice. Another strength of this study is the wide spectrum of dementia subtypes that have been distinguished in SveDem. Such detailed information is rarely encountered in studies assessing survival in dementia, and when this information is available, groups of patients with less common dementia types are often too small for meaningful subgroup analyses. Although our study population accurately reflects the Swedish population with a clinical diagnosis of dementia, it should be noted that dementia is generally known to be severely underdiagnosed.^21^ According to population-based studies, SveDem is estimated to cover ≈38% of the entire population with dementia in Sweden.^22^ The ascertainment of dates of death, medical diagnoses, and medications through national registers allowed complete follow-up and eliminated the risk of attrition and recall bias. The individuals excluded because of missing values for MMSE, dementia type, or living situation differed slightly from the study population in terms of survival, cognition, and medication use, which may have introduced selection bias. However, sensitivity analyses with worst-case and best-case scenarios indicated that the potential impact of selection bias is limited because the number of excluded individuals was <10% of our population. Although the Swedish National Patient Registry has >99% coverage of inpatient medical diagnoses, the primary care diagnoses are lacking, causing the prevalence of diseases such as diabetes mellitus (which is often diagnosed in primary care) to be underestimated. The effect of the CCI score observed in the present study may therefore also be underestimated. Unfortunately, more detailed information on a patient's physical health such as the severity of comorbidity, the number of hospitalizations, or the patient's frailty status was unavailable in SveDem. In addition, information on genetic factors and educational attainment was unavailable in this registry-based cohort. The medication data from the Swedish Prescribed Drug Register provide 100% coverage for all medications taken out of the pharmacy but do not include over-the-counter medication and medications given during hospital admission. Because the type of medication available over the counter is limited in Sweden, we do not expect this to have caused substantial bias.

Previous studies have predominantly emphasized the influence of single characteristics on survival. For example, lower cognitive performance at the time of diagnosis appears to be related to an increased mortality risk in dementia in several studies.^23,24^ A Swedish population study showed that living alone shortened survival by 0.6 years among older people.^25^ A 15-year follow-up study of people with dementia treated with cholinesterase inhibitors showed that impaired daily functioning and greater number of medications are associated with shorter survival after AD diagnosis.^26^ Other previously reported risk factors for mortality in older people with and without dementia are increased frailty^27,28^ and multimorbidity.^29^ Previous studies based on SveDem data also showed an association between dementia subtypes other than AD and mortality,^20^ as well as an association between mortality and low body mass index.^30^ Despite these extensive examinations of risk factors for mortality in dementia, the prognostic performance of multivariable prediction models for survival in dementia is rarely studied. In the present study, we used SveDem data to evaluate the performance (i.e., prognostic value) of a prediction model for survival based on routinely collected data. Our models had good predictive accuracy, as shown by the calibration plots, and the discriminative ability of the models appeared to be modest yet comparable to that of other clinical prediction models such as the Framingham Coronary Heart Disease Score.^31,32^ The high shrinkage factors and low optimism in the bootstrapped Harrell c index also indicated that our developed models perform well in Swedish people diagnosed with dementia. We expect our findings to be fairly generalizable to people with dementia in other Western European countries, especially countries with similar population structures and health care systems. However, variations in population age, education levels, and ethnic composition across countries may decrease the performance of our model. Therefore, we recommend recalibrating the prediction model before applying it in other settings because our model has not yet been externally validated. Moreover, to provide a concise tool, we decided to include in our risk tables only those predictors found to be most important in our sample. The predictors that were not included in our risk tables, i.e., living situation and number of drugs, may have considerably more predictive value in other populations. Hence, external validation is important, and future studies may benefit from including all variables listed in table 3 because all were found to be significant predictors of survival.

In today's care system, shared decision-making for the planning of care and end-of-life decisions for persons with dementia and their caregivers is becoming increasingly important. With the rising prevalence of dementia, the need to provide personalized prognosis for dementia also is growing. For these reasons, it is more important than ever for clinicians to be able to provide accurate information on estimated survival. This study provides a practical tool to predict survival of people with dementia and thereby aid shared decision-making and advance care planning for this rapidly growing patient group. Moreover, this study provides estimates of median life expectancy for patients with dementia, stratified by patient characteristics. This type of detailed reference data is scarce is literature. We acknowledge that there are substantial barriers to providing numerical estimates of life expectancy to patients^33^; both underestimation and overestimation of survival time may have a negative impact on patients and their families. For this reason, we presented our results in the form of a color scheme representing 3-year survival probabilities, opposed to an estimate of remaining survival time used in a previously developed tool.^34^ The risk tables presented in this study may help physicians incorporate patients' life expectancy into clinical decision-making. Rather than raising the (false) belief that an accurate estimate of remaining survival time can be given, the colors are intended to communicate an increasing urgency to consider advance care planning. Thus, the visual representation of 3-year survival probabilities may aid physicians in addressing the sensitive topic of end of life in conversations with patients and caregivers. This may be particularly important for patients at high risk of mortality who experience acute events that require possibly harmful interventions because these patients may decide to refuse treatment in favor of quality of life.^35^ Informing patients and their families in a timely manner about their prognosis will also allow them to make the arrangements needed to anticipate events associated with end of life such as nursing home placement.

This study showed that it is possible to stratify individuals with dementia into groups with distinct survival probabilities based on 5 easily obtainable variables. By providing more insight into patients' life expectancy, the developed risk tables may aid shared decision-making and advance care planning in dementia. Future studies should focus on determining the predictive performance of the model in other settings (i.e., external validity) and evaluating the impact of its implementation in clinical practice.^36^

## Glossary

AD: Alzheimer disease
CCI: Charlson Comorbidity Index
CI: confidence interval
HR: hazard ratio
ICD-10: *International Classification of Diseases, 10 revision*
IQR: interquartile range
MMSE: Mini-Mental State Examination
SveDem: Svenska Demensregistret
